# Efficacy of continuous erythropoietin receptor activator for end-stage renal disease patients with renal anemia before and after peritoneal dialysis initiation

**DOI:** 10.1007/s10157-020-01973-x

**Published:** 2020-10-06

**Authors:** Daisuke Fujimoto, Masataka Adachi, Yoshikazu Miyasato, Yusuke Hata, Hideki Inoue, Akira Oda, Yutaka Kakizoe, Terumasa Nakagawa, Akiko Shimasaki, Keishi Nakamura, Yu Nagayoshi, Masashi Mukoyama

**Affiliations:** Department of Nephrology, Kumamoto University Graduate School of Medical Sciences, 1-1-1 Honjo, Chuo-ku, Kumamoto, 860-8556 Japan

**Keywords:** Continuous erythropoietin receptor activator (CERA), Diabetes mellitus, Erythropoietin resistance index (ERI), Peritoneal dialysis, Renal anemia

## Abstract

**Background:**

Serial management of renal anemia using continuous erythropoietin receptor activator (CERA) throughout the peritoneal dialysis initiation period has rarely been reported. We investigated the efficacy and dosage of CERA treatment from pre- to post-peritoneal dialysis initiation for anemia management in patients with end-stage renal disease.

**Methods:**

Twenty-six patients (13 men; mean age 60.9 years) who started peritoneal dialysis between April 2012 and April 2018 were investigated. Serial changes in hemoglobin levels, transferrin saturation and ferritin levels, CERA dosage, and the erythropoietin resistance index (ERI) over a 48 week period were retrospectively examined.

**Results:**

Mean hemoglobin levels increased significantly from 10.5 g/dL at 24 weeks prior to the peritoneal dialysis initiation to 11.5 g/dL at 4 weeks post-initiation. The proportion of patients with hemoglobin levels ≥ 11 g/dL increased significantly after peritoneal dialysis initiation. The mean CERA dosage was 57.0 µg/month at 24 weeks prior to dialysis initiation, 86.5 µg/month at initiation, and 72.0 µg/month at 4 weeks post-initiation. Thus, the dosage tended to increase immediately before peritoneal dialysis initiation and then decreased thereafter. Hemoglobin levels were significantly lower, while the CERA dosage for maintaining hemoglobin levels and ERI tended to be higher at dialysis initiation in patients with diabetes than in those without diabetes.

**Conclusion:**

Treatment with CERA prior to and during the peritoneal dialysis initiation achieved fairly good anemia management in patients with and without diabetes. The CERA dosage could be reduced in patients without diabetes after dialysis initiation.

**Electronic supplementary material:**

The online version of this article (10.1007/s10157-020-01973-x) contains supplementary material, which is available to authorized users.

## Introduction

Anemia is an almost inevitable complication of end-stage renal disease (ESRD) due to the inadequate production of endogenous erythropoietin by the impaired kidneys [1]. Renal anemia is significantly associated with cardiovascular events and all-cause mortality [2, 3]. Erythropoiesis-stimulating agents (ESAs) improve not only renal anemia but also the related clinical outcomes, including better quality of life (QOL) [4], longer kidney survival [5, 6], and slower progression of left ventricular hypertrophy (LVH) in patients with chronic kidney disease (CKD) [7, 8]. Current guidelines recommend a target hemoglobin (Hb) level of 10.0–12.0 g/dL for dialysis and 10.0–13.0 g/dL for non-dialysis CKD patients [9–11].

Continuous erythropoietin receptor activator (CERA) is a chemically synthesized ESA that differs from epoetin beta through the integration of an amide bond between an amino group of epoetin beta and a specific, linear methoxy-polyethylene glycol polymer chain [12]. When administered intravenously or subcutaneously, CERA exhibits a long half-life (approximately 130 h) and low clearance; these characteristics, together with its unique receptor-binding properties, feature a novel pharmacological profile different from that of other ESAs currently used [12–14].

To date, many studies on the treatment for renal anemia using ESAs have been reported in peritoneal dialysis (PD) patients before and after dialysis initiation [14–16]. However, there have been few reports on the serial management of renal anemia using CERA throughout the period from pre- to post-PD initiation. Furthermore, it has been shown that patients with diabetic kidney disease, a major cause of ESRD, develop renal anemia earlier than those with other types of renal diseases [17], but information about the comparison of anemia management during a PD initiation period is scarce.

Therefore, we here investigated the usefulness of CERA for anemia management in PD patients, to examine its efficacy and dosage before and after PD initiation. We also investigated the impact of the presence of diabetes on anemia control in PD patients, with a view to evaluating the treatment efficacy of CERA.

## Subjects and methods

### Participants and study design

**Fig. 1:**
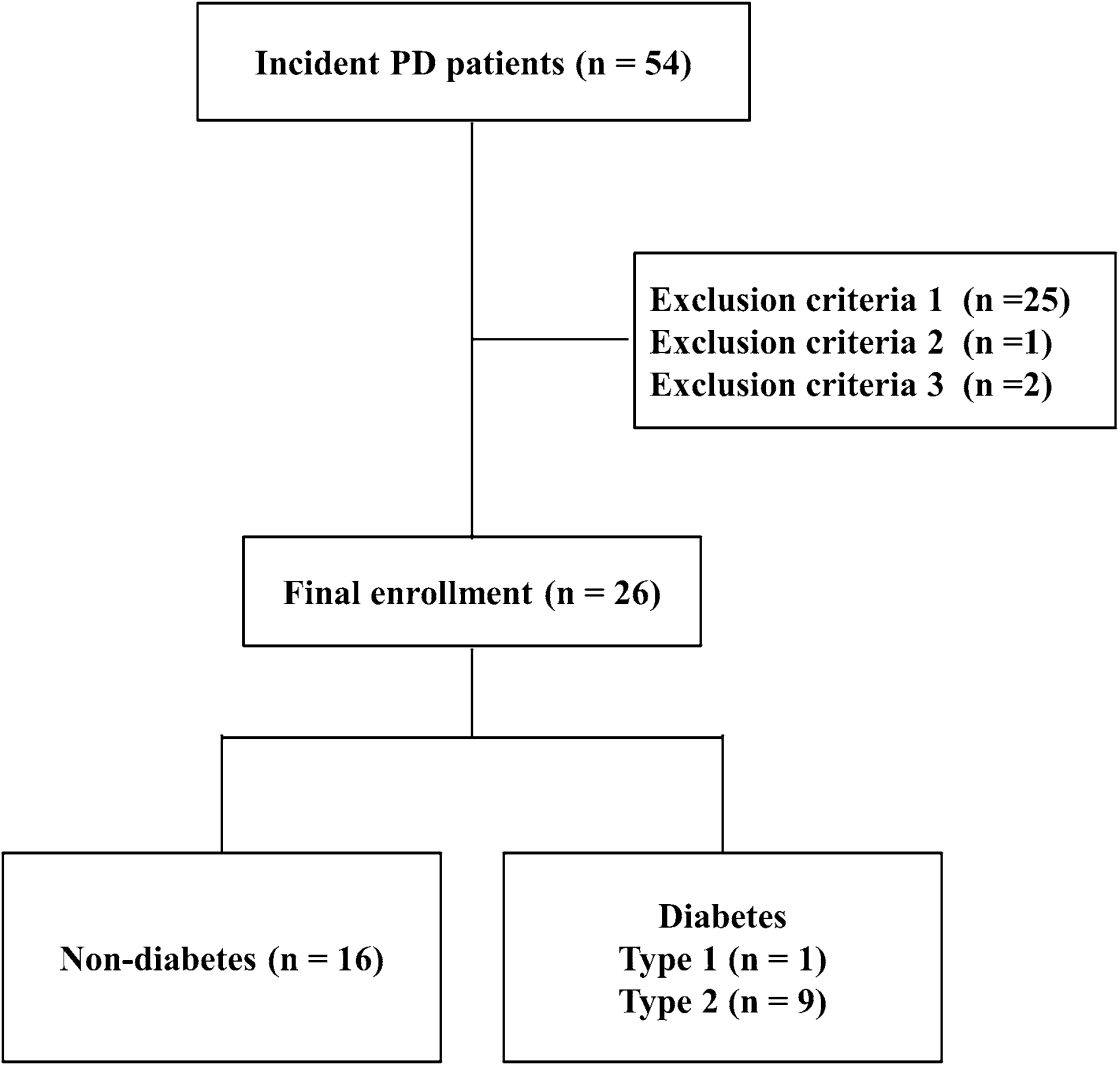
A flow-chart showing the screening and enrollment of the patients

Data from 26 patients with ESRD, who were older than 18 years and who started PD between April 2012 and April 2018 at Kumamoto University Hospital, Japan, were analyzed in this study. Patients were receiving maintenance continuous ambulatory peritoneal dialysis (CAPD), tidal peritoneal dialysis (TPD), or nightly peritoneal dialysis (NPD). Exclusion criteria were as follows: (1) treatment with recombinant human erythropoietin or ESAs other than CERA, (2) blood transfusion therapy during the observational period, and (3) known malignancies (Fig. 1).

This study was a retrospective, single-center observational survey, performed using an opt-out method of enrolment at Kumamoto University Hospital.

### Laboratory measurements and data collection

The serum for determination of biochemical parameters was obtained from each patient at regular outpatient visits. Hb levels, iron parameters [transferrin saturation (TSAT) and ferritin], serum albumin (Alb), serum creatinine (Cr), C-reactive protein (CRP), and intact parathyroid hormone (iPTH) levels were determined once a month at SRL, Inc. (Tokyo, Japan).

The estimated glomerular filtration rate (eGFR) of each participant was calculated using the following formula [18]:

$${\text{eGFR }}\left( {{\text{mL}}/{\min}/{1}.{\text{73 m}}^{{2}} } \right)\, = \,{194}\, \times \,{\text{serum creatinine}}^{{ - {1}.0{94}}} \, \times \,{\text{age}}^{{ - \;0.{287}}} \, \times \,0.{739}\;({\text{if female}})$$

iPTH was measured by chemiluminescent immunoassay. Baseline demographic and clinical characteristics, including age, sex, body weight, co-morbidities, laboratory data, cardiothoracic ratio, and iron preparation use were recorded.

### Assessments

Hb levels, iron parameters, CERA dosage, and erythropoietin resistance index (ERI) [CERA amount (µg)/body weight (kg)/Hb (g/dL)/4 weeks] were monitored retrospectively over a period of 48 weeks, from 24 weeks before to 24 weeks after PD initiation. We also compared the differences in these parameters between patients with and without diabetes (10 and 16 patients, respectively).

### Statistical analyses

Statistical analyses were performed using JMP statistical discovery software, version 11.0 (SAS Institute Inc., Cary, NC, USA) for Windows. Data are expressed as mean ± SD for normally distributed (parametric) continuous variables.

For efficacy analyses, we compared the mean change in Hb level, CERA dosage, iron parameters, and ERI between the baseline and other time-points for patients who received CERA, using a one-way analysis of variance. McNemar’s test was used to calculate the changes in Hb level categories (Hb levels ≥ or < 11 g/dL) before and after PD initiation. Comparisons between patients with and without diabetes were performed using Welch’s *t *test. *P* < 0.05 was considered statistically significant.

## Results

Table 1 shows the baseline demographic and laboratory data of the patients enrolled at the initiation of PD therapy. Thirteen men and 13 women with a mean age of 60.9 ± 11.7 years were included. All patients received adequate dialysis treatment. The most common cause of ESRD was diabetic kidney disease (38.4%), followed by IgA nephropathy (26.9%), and hypertensive nephrosclerosis (11.5%). Mean Hb levels were 10.1 ± 1.1 g/dL; TSAT and serum ferritin levels were adequately controlled (TSAT 25.2 ± 8.3%, ferritin 146.1 ± 134.1 ng/mL). There were no active infectious diseases, severe heart failure, or gastrointestinal bleeding during the evaluation period. The percentage of patients receiving iron replacement therapy was 11.5%. Renin-angiotensin system (RAS) inhibitors [angiotensin-converting-enzyme inhibitor (ACEI) and angiotensin II receptor blocker (ARB)] were administered to 19 (73.1%) patients at PD initiation. Hb levels between groups with or without RAS inhibitors were not significantly different [average Hb level 10.1 g/dL (with RAS inhibitor) vs. 9.8 g/dL (without RAS inhibitor), *P* = 0.252]. No adverse events were observed during this study.

**Table 1:** Baseline demographics and clinical characteristics of the patients

Patient number	26 (male/female = 13/13)
Age (years)	60.9 ± 11.7
Body weight (kg)	60.8 ± 9.9
Cause of CKD, *n* (%)	
Diabetic kidney disease	10 (38.4)
IgA nephropathy	7 (26.9)
Hypertensive nephrosclerosis	3 (11.5)
ADPKD	1 (3.8)
FSGS	1 (3.8)
Lupus nephritis	1 (3.8)
Others (or unknown)	3 (11.5)
BUN (mg/dL)	74.7 ± 25.1
Serum Cr (mg/dL)	9.47 ± 1.90
eGFR (mL/min/1.73 m^2^)	4.65 ± 1.35
Hb (g/dL)	10.1 ± 1.1
TSAT (%)	25.2 ± 8.3
Ferritin (ng/mL)	146.1 ± 134.1
Serum Alb (g/dL)	3.5 ± 0.5
Intact PTH (pg/mL)	263.6 ± 170.9
CRP (mg/dL)	0.28 ± 0.38
BNP (pg/mL)	165.1 ± 454.7
Cardiothoracic ratio (%)	49.5 ± 5.41
Iron preparation use	11.5% (3/26)
Use of RAS inhibitor (ACE inhibitor or ARB)	73.1% (19/26)

Values expressed as means ± SD

*ADPKD* Autosomal dominant polycystic kidney disease, *FSGS* Focal segmental glomerulosclerosis, *BUN* blood urea nitrogen, *Cr* creatinine, *eGFR* estimated glomerular filtration rate, *Hb* hemoglobin, *TSAT* transferrin saturation, *Alb* albumin, *PTH* parathyroid hormone, *CRP* C-reactive protein, *BNP* brain (or B-type) natriuretic peptide, *RAS* renin‒angiotensin system, *ACE inhibitor* angiotensin-converting enzyme inhibitor, *ARB* angiotensin II receptor blocker

The PD therapy varied. Nineteen patients were treated using CAPD, 3 patients were treated by TPD, and 4 patients by NPD.

**Fig. 2:**
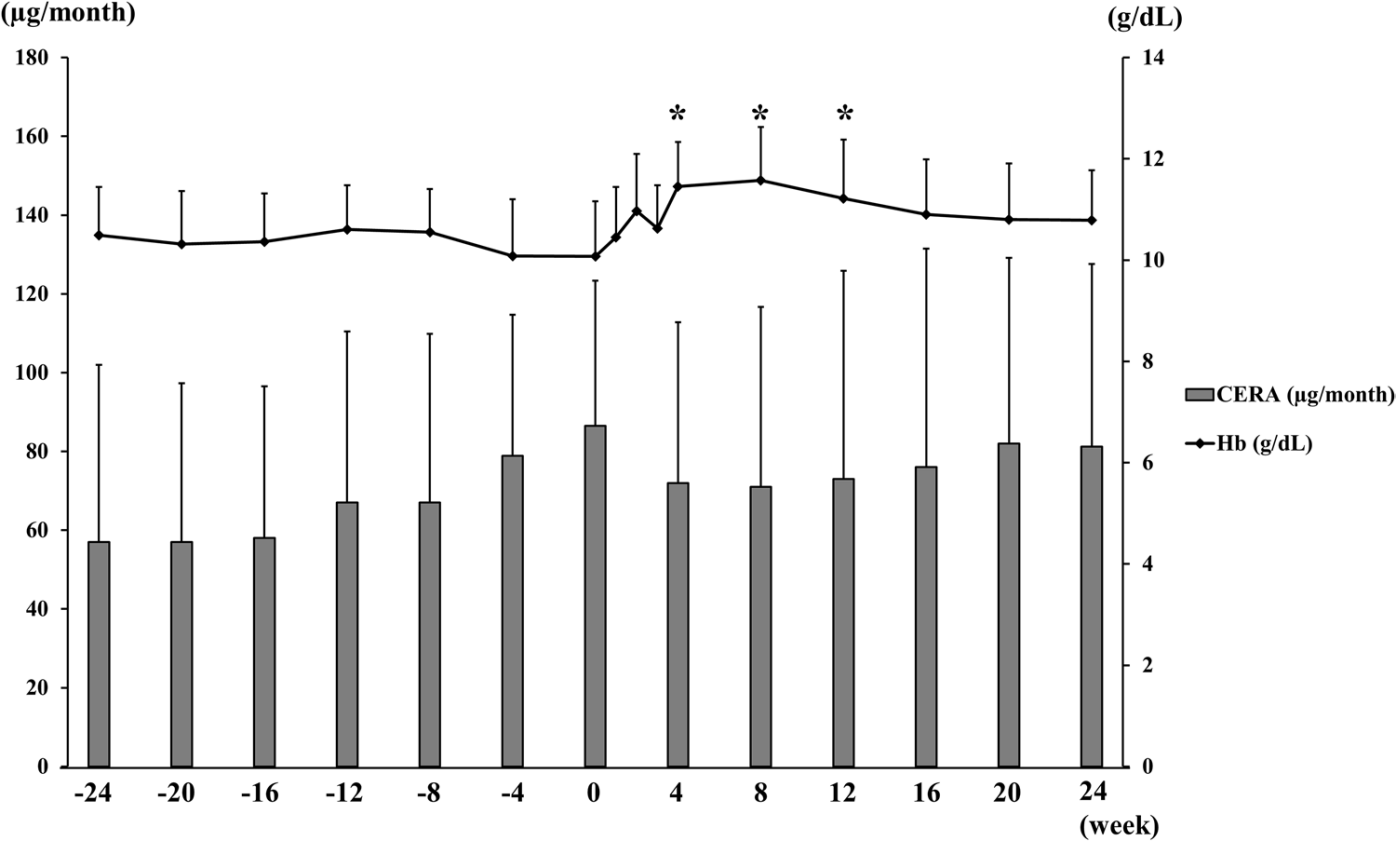
Serial changes in Hb levels and CERA dosage from 24 weeks before through 24 weeks after PD initiation. Values are expressed as means ± SD. Left axis: dosage of CERA (μg/month), Right axis: Hb level (g/dL) *P < 0.05 vs. week 0. *Hb* hemoglobin, *CERA* continuous erythropoietin receptor activator,* PD* peritoneal dialysis

The mean Hb levels were 10.5 g/dL at 24 weeks prior to PD initiation, 10.1 g/dL at PD initiation, and 11.5 g/dL at 4 weeks after initiation (Fig. 2). Thus, time-dependent changes revealed that Hb levels tended to decrease as PD initiation approached, reached their lowest levels at the time of PD initiation, and significantly increased after PD initiation (at weeks 4, 8, and 12 after initiation) (Fig. 2). Throughout this period, the average Hb levels rarely fell below 10 g/dL. The mean CERA dosage was 57.0 µg/month at 24 weeks prior to PD initiation, 86.5 µg/month at PD initiation, and 72.0 µg/month at 4 weeks after initiation (Fig. 2). Thus, CERA dosage tended to increase immediately before PD initiation and then tended to decrease thereafter.

**Fig. 3:**
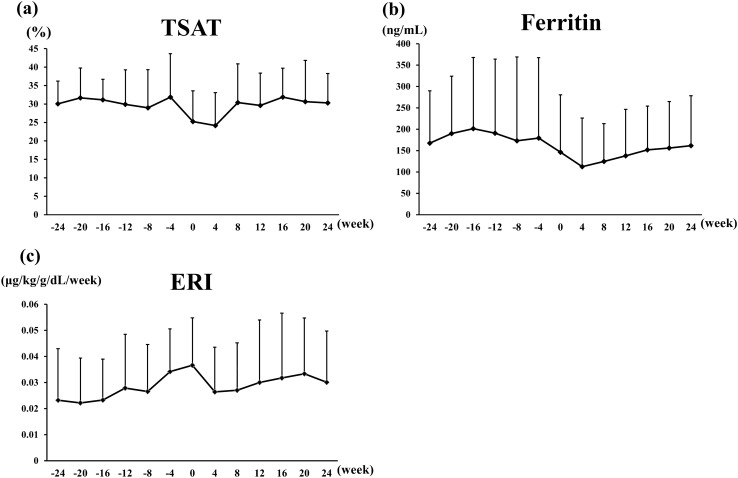
Changes in TSAT (**a**), ferritin levels (**b**), and ESA resistance index (**c**) from 24 weeks before through 24 weeks after PD initiation. Values are expressed as means ± SD. *TSAT* transferrin saturation, *PD* peritoneal dialysis, *ESA* erythropoiesis-stimulating agents, *ERI* erythropoietin resistance index. ERI is calculated as follows: ERI = CERA dosage (μg/month)/BW (kg)/Hb (g/dL)/4 weeks

Figures 3a, b show the changes in TSAT and ferritin levels. Throughout the observation period, average TSAT levels were maintained above 20%, suggesting that iron levels were sufficient. Three of the enrolled patients received iron-replacement therapy; during the course, no significant differences in the iron-related data were observed between these patients and patients who did not receive iron replacement. Serum ferritin levels tended to be higher prior to PD initiation than after initiation. ERI, an indicator of hypo-responsiveness to ESA treatment, tended to increase as PD initiation approached, peaked at PD initiation, and tended to decrease after PD initiation (Fig. 3c). ERI gradually increased again over time after PD initiation.

We evaluated the changes in the number of patients with Hb levels exceeding the target value of 11 g/dL before and after PD initiation. Table 2 shows the results analyzed by McNemar’s test. After PD initiation (at weeks 4 and 8 after initiation), the number of patients with Hb levels above 11 g/dL significantly increased, as compared to the number at 4 weeks before PD initiation.

**Table 2:** The 2 × 2 tables showing the number of patients with Hb levels ≥ 11 g/dL or < 11 g/dL before and after PD initiation

(a) Comparison between − 4 weeks vs. 4 weeks				
− 4 w vs. 4 w		Week 4 (after initiation)		
		Hb ≥ 11	Hb < 11	
Week − 4 (before)	Hb ≥ 11	3	1	McNemar’s test
	Hb < 11	14	7	*P* < 0.05

(b) Comparison between − 4 weeks vs. 8 weeks				
− 4 w vs. 8 w		Week 8 (after initiation)		
		Hb ≥ 11	Hb < 11	
Week − 4 (before)	Hb ≥ 11	3	1	McNemar’s test
	Hb < 11	16	5	*P* < 0.05

*P *values were calculated using statistical analysis of McNemar’s test

*Hb* hemoglobin

Next, we compared the data of patients with and without diabetes (10 and 16 each) in order to examine whether diabetes affects the efficacy of CERA for anemia management. Table 3 shows the data of these patient groups at PD initiation. There were no significant differences between the two groups in terms of age, body weight, blood urea nitrogen, serum Cr, serum albumin, iPTH, CRP, brain natriuretic peptide, or HbA1c at PD initiation.

**Table 3:** Comparison of the data at PD initiation between the patients without diabetes (Non-DM) and those with diabetes (DM)

	Non-DM	DM	*P *value
Patient number	16	10	
Age (years)	63.4 ± 9.8	57.1 ± 13.4	0.12 (NS)
Sex	male/female = 7/9	male/female = 6/4	
Body weight (kg)	59.3 ± 8.6	63.3 ± 11.3	0.18 (NS)
BUN (mg/dL)	75.2 ± 24.1	73.9 ± 26.7	0.44 (NS)
Serum Cr (mg/dL)	9.06 ± 1.47	10.13 ± 2.29	0.11 (NS)
eGFR (mL/min/1.73 m^2^)	4.63 ± 1.16	4.70 ± 1.61	0.45 (NS)
Serum Alb (g/dL)	3.51 ± 0.37	3.44 ± 0.63	0.37 (NS)
Intact PTH (pg/mL)	300.7 ± 185.7	208.1 ± 127.2	0.08 (NS)
CRP (mg/dL)	0.34 ± 0.43	0.19 ± 0.28	0.33 (NS)
BNP (pg/mL)	199.4 ± 574.3	110.3 ± 70.6	0.28 (NS)
HbA1c (%)	5.6 ± 0.4	6.1 ± 1.2	0.12 (NS)

Values are expressed as means ± SD. *P* values are calculated using Welch’s *t *test

*BUN* serum blood urea nitrogen, *Cr* creatinine, *eGFR* estimated glomerular filtration rate, *Alb* albumin, *PTH* parathyroid hormone, *CRP* C-reactive protein, *BNP* brain (or B-type) natriuretic peptide, *NS* not significant

**Fig. 4:**
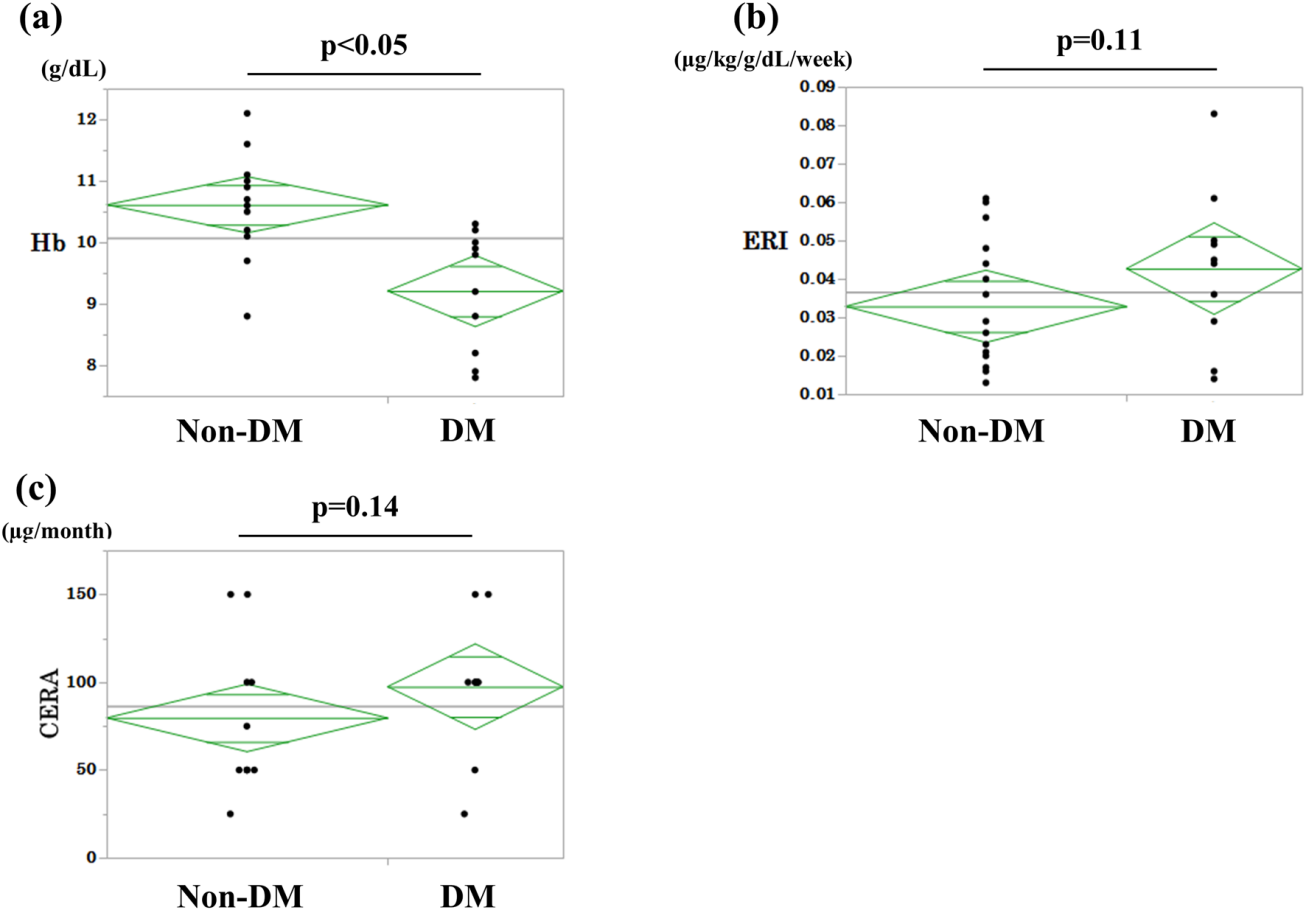
Comparison of Hb levels (**a**), ERI (**b**), and CERA dosage (**c**) at PD initiation among patients with diabetes vs. patients without diabetes. *Hb* hemoglobin, *CERA* continuous erythropoietin receptor activator, *PD* peritoneal dialysis, *ERI* erythropoietin resistance index, *ESA* erythropoiesis-stimulating agents, *Non-DM* non-diabetes, *DM* diabetes. Analyses were performed using Welch’s *t* test. Patients with diabetes: *n* = 10; patients without diabetes: *n* = 16

Figure 4 shows the comparison of Hb levels (Fig. 4a), ERI (Fig. 4b), and CERA dosage (Fig. 4c) between the patients with and without diabetes at the time of PD initiation. The average Hb level of patients without diabetes was 10.6 ± 0.8 g/dL, while that of patients with diabetes was 9.2 ± 0.9 g/dL; the Hb level was significantly lower in the group with diabetes. The average ERI values were 0.033 ± 0.016 and 0.042 ± 0.019 in the groups without and with diabetes, respectively, and thus tended to be higher in patients with diabetes. The average CERA dosages were 79.7 ± 35.6 µg/month and 97.2 ± 38.1 µg/month in patients without and with diabetes, respectively, showing a trend for a higher dosage in those with diabetes.

**Fig. 5:**
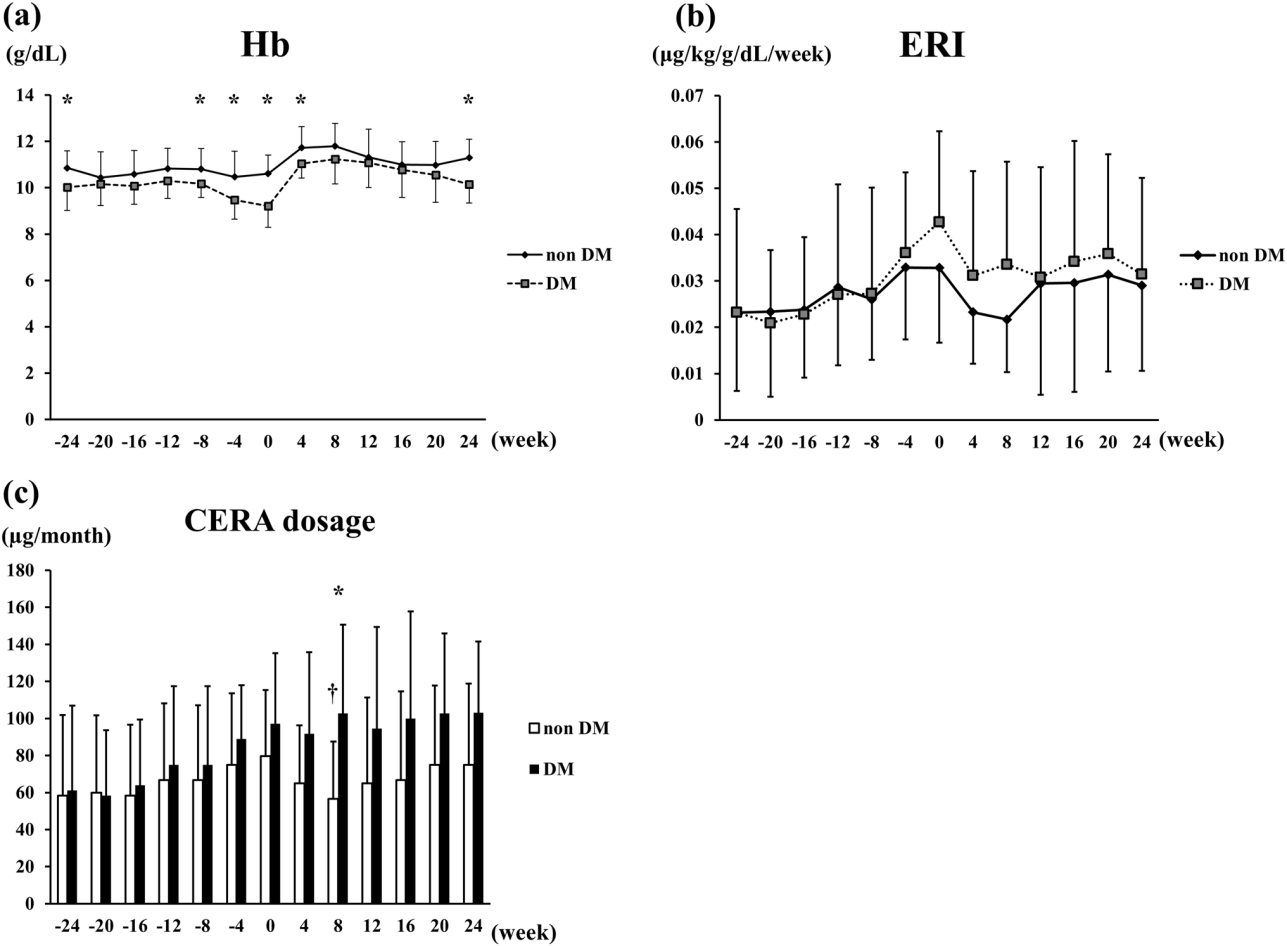
Comparison of Hb levels (**a**), ERI (**b**), and CERA dosage (**c**) from 24 weeks before through 24 weeks after PD initiation between patients with and without diabetes. *Hb* hemoglobin, *CERA* continuous erythropoietin receptor activator, *PD* peritoneal dialysis, *ERI* erythropoietin resistance index, *ESA* erythropoiesis-stimulating agents. Analyses were done by Welch’s *t* test. * < 0.05 (DM vs. non-DM), † < 0.05 (week 8 vs. week 0)

Figure 5 shows the chronological changes in Hb levels (Fig. 5a), ERI (Fig. 5b), and CERA dosage (Fig. 5c) in the patients with and without diabetes throughout the evaluation period. Hb levels appeared to be higher in the group without diabetes during the period from pre- to post-PD initiation, while CERA dosage and ERI tended to be lower for most of this period. Of note, in the group without diabetes, CERA dosage significantly decreased after PD initiation (79.7–56.7 µg at 8 weeks), whereas no change was observed in the group with diabetes (97.2–102.8 µg at 8 weeks). There were no adverse reactions to CERA throughout the evaluation period.

## Discussion

In the care of patients with advanced CKD, there is an increasing awareness of the importance of the optimal transition from the pre-dialysis run-up to renal replacement therapy, including PD [19]. In particular, the adequate management of anemia is reported to be critical in the incident hemodialysis (HD) patients for preventing cardio- and cerebrovascular events during this period [20]. It has been proposed that treatment using CERA could achieve successful anemia control in patients on maintenance PD [21]; however, very few reports have examined anemia management by ESA throughout the period from pre- to post-PD initiation. We, therefore, assessed the effect of CERA treatment during the period from pre- to post-PD initiation and compared the efficacy of treatment in patients with and without diabetes.

In the present study, we showed that Hb levels tended to decrease as PD initiation approached, and then increased after PD initiation. This trend was also previously observed in HD patients [20]. Several studies have suggested that uremic toxins or uremia-induced inhibitors of erythropoiesis could contribute to the aggravation of renal anemia [22, 23]. Our results suggested that mitigating uremic status by initiating dialysis could improve renal anemia without the reinforcement of ESA treatment. This notion was also consistent with the apparent decrease of ERI after PD initiation (Fig. 3). However, our study involved the periods from the last CERA administration just prior to PD initiation up to PD initiation and from PD initiation up to the next CERA administration, which varied across patients (without a 4-week interval). Therefore, the elevation of Hb levels might be partly due to the CERA dosage increase just before PD initiation.

Our data showed that Hb measurements returned to low levels at 24 weeks after PD initiation. We assumed that this finding was due to the decreased residual renal function. Data on residual renal function at 24 weeks after PD initiation were not available; however, we routinely evaluated daily urine volume, residual renal Kt/V urea, and creatinine clearance (Ccr) at 1 year after PD initiation. As shown in Supplementary Fig. S1 and Supplementary Table S1, residual renal function significantly decreased after 1 year; therefore, we presumed that residual renal function decreased to a certain extent at 24 weeks after PD initiation.

The target Hb level for patients with CKD without dialysis and those undergoing PD is between 11 g/dL to 13 g/dL, according to the guidelines of the Japanese Society for Dialysis Therapy [9]. In this study, the average Hb levels did not fall below 10 g/dL throughout the evaluation period. In the post-PD initiation period in particular, the percentage of patients with Hb levels above 11 g/dL increased significantly. These results indicate that anemia treatment using CERA throughout the period from pre- to post-PD initiation is useful for maintaining the target Hb level. To our knowledge, assessment of renal anemia using CERA throughout the period from pre- to post-PD initiation has not been reported to date. However, during the period without dialysis, more than half of the CKD patients could not reach the target Hb level. We speculate that a portion of patients failed to receive an adequate ESA treatment before PD initiation, partly because of its cost and infrequent hospital visits during the non-dialysis period. In Japan, during this period, patients have to pay 10–30% of their medical expenses themselves; patients receiving dialysis can obtain more financial support than non-dialysis patients.

There were several notable differences between previous reports of HD patients receiving CERA treatment and the current study. The average CERA dosage tended to be less, and Hb levels tended to be higher in our patients than in those who started HD [24, 25]. PD is often initiated from an earlier stage of renal failure than is HD, because the residual renal function is critical for optimally performing and continuing PD therapy. Under such circumstances, interstitial damage is assumed to be milder, so that relatively high Hb levels can be maintained.

Patients with diabetic nephropathy were reported to develop renal anemia from an earlier stage of renal failure than did those with other causes of CKD [17]. To date, few reports have examined the association of diabetes with ESA treatment in patients receiving PD. In HD patients or non-dialysis CKD patients, several investigations have compared patients with and without diabetes [26–28]; however, the conclusions were controversial. In the present study, Hb levels were significantly lower in patients with diabetes, although other characteristics were not significantly different. CERA dosage and ERI tended to be higher in the group with diabetes. Moreover, in patients without diabetes, CERA dosage decreased significantly after PD initiation, whereas it remained unchanged in patients with diabetes. These results suggested that the presence of diabetes could have a negative influence on renal anemia management. Chronic inflammation has been reported to be involved in the progression of diabetic kidney disease, including tubulointerstitial lesions [29, 30], and this may be associated with deterioration of renal anemia and possible ESA hypo-responsiveness in patients with diabetes. Moreover, the residual renal function of diabetic patients tended to decline faster than that of non-diabetic patients (the decline of Ccr during 1 year after PD initiation was 32.8 ± 14.7 L/week in diabetic patients and 21.1 ± 24.5 L/week in non-diabetic patients, which was not significantly different), and two of 10 diabetic patients were forced to stop PD, while none of the non-diabetic patients stopped within 1 year after PD initiation. These differences may be associated with the higher dose of CERA and lower Hb levels in diabetic patients after PD initiation.

Other than diabetes, factors that may influence anemia are excess body fluid (and/or congestive heart failure) and RAS inhibition. The chronological change in BNP after PD initiation is shown in Supplementary Fig. S2. We had insufficient data for prior to PD initiation. The values varied widely, and no significant correlations with anemia parameters were observed. RAS inhibition is reported to be associated with renal anemia progression, because it may suppress erythropoietin production [31]. We evaluated the average Hb levels in patients with or without RAS inhibitor treatment. The average Hb levels were 10.1 g/dL in patients with RAS inhibitors and 9.8 g/dL in patients without RAS inhibitors, which was not significantly different.

There were several limitations to our study. First, the sample size was small; therefore, the statistical power was insufficient to detect any statistical significance in the correlation between two or more variables. Second, the observational period was only 48 weeks, and we could not evaluate the long-term outcomes and prognosis. Several studies have reported that ESA responsiveness is associated with long-term prognosis in HD patients or renal survival in CKD patients [32, 33]. Hence, follow-up of these PD patients for a more extended period could provide further insights.

In conclusion, CERA was shown to be effective for adequate anemia management, both before and after PD initiation. Moreover, the results suggested that the required dosage of CERA could be reduced in patients without diabetes after PD initiation, whereas the dose may remain unchanged in those with diabetes. Further investigation with a larger number of patients is warranted to validate this notion in the future.

## Electronic supplementary material

Below is the link to the electronic supplementary material.

Supplementary file1 (DOCX 14 kb)

Supplementary file2 (TIF 936 kb)
