# Supplementary material for: Efficacy of continuous erythropoietin receptor activator for end-stage renal disease patients with renal anemia before and after peritoneal dialysis initiation
Source: Clin Exp Nephrol. 2020 Oct 6;25(2):191–9. doi: 10.1007/s10157-020-01973-x (PMC7880977; doi:10.1007/s10157-020-01973-x)
Supplement: Supplementary file 1 — Supplementary file1 (DOCX 14 kb) [file 10157_2020_1973_MOESM1_ESM.docx]

**Supplementary Table S1. Changes in the parameters of residual kidney function at the point of PD initiation and 1 year after PD initiation.**

|  | **PD initiation** | **After 1 year** | **P-value** |
| --- | --- | --- | --- |
| Urine volume (mL/day) | 1165.2 ± 295.8 | 890.9 ± 405.5 | 0.007 |
| Weekly Kt/V | 0.82 ± 0.27 | 0.59 ± 0.30 | 0.027 |
| Ccr of kidney (L/week) | 60.0 ± 17.2 | 35.0 ± 22.0 | 0.030 |

Values are expressed as means ± SD. P values are calculated using Welch’s *t*-test.

Ccr, Creatinine clearance.
