# Supplementary figures and images for: Efficacy of continuous erythropoietin receptor activator for end-stage renal disease patients with renal anemia before and after peritoneal dialysis initiation
Source: Clin Exp Nephrol. 2020 Oct 6;25(2):191–9. doi: 10.1007/s10157-020-01973-x (PMC7880977; doi:10.1007/s10157-020-01973-x)

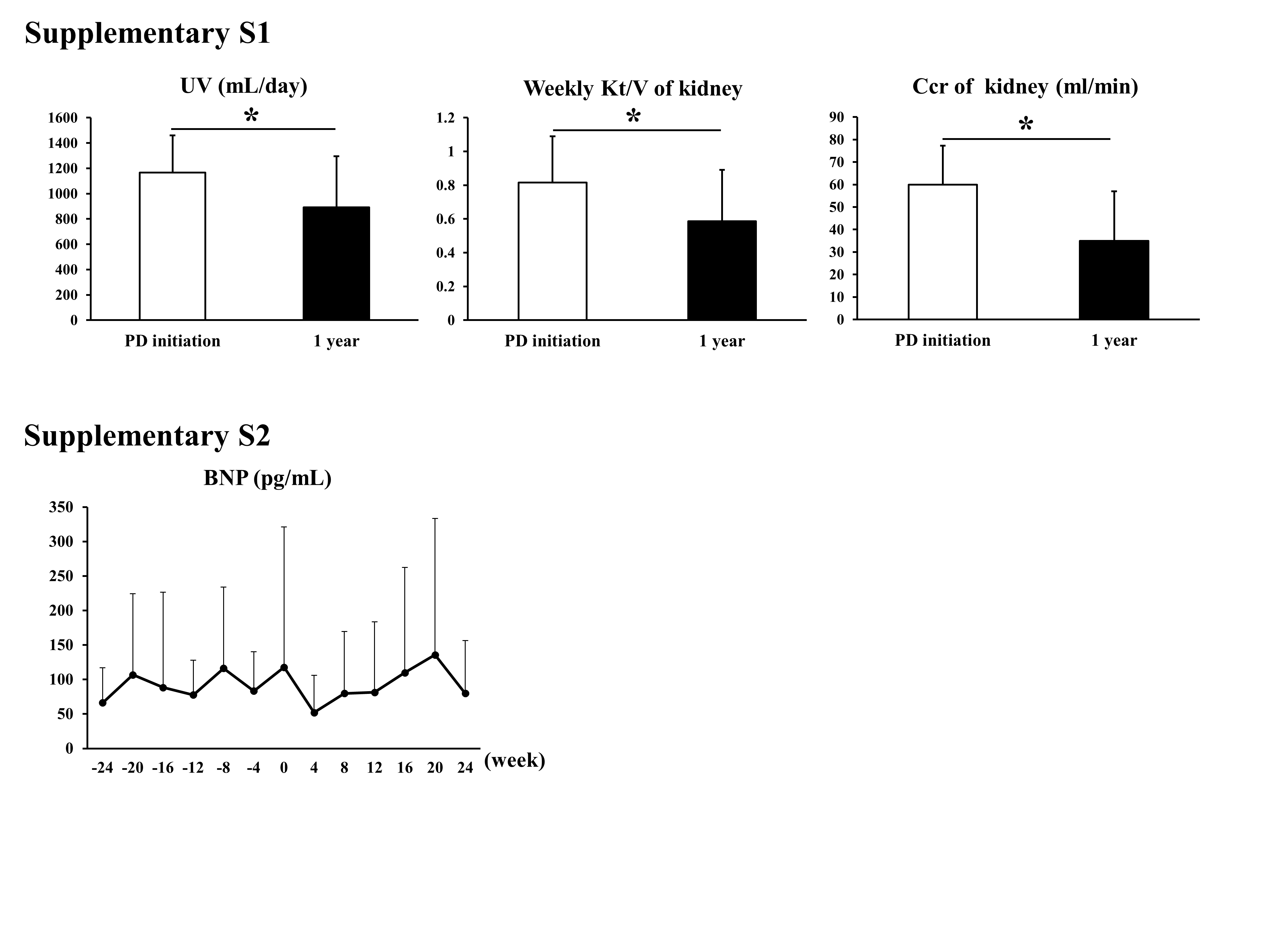

Supplement: Supplementary file 2 — Supplementary file2 (TIF 936 kb) [file 10157_2020_1973_MOESM2_ESM.tif]
